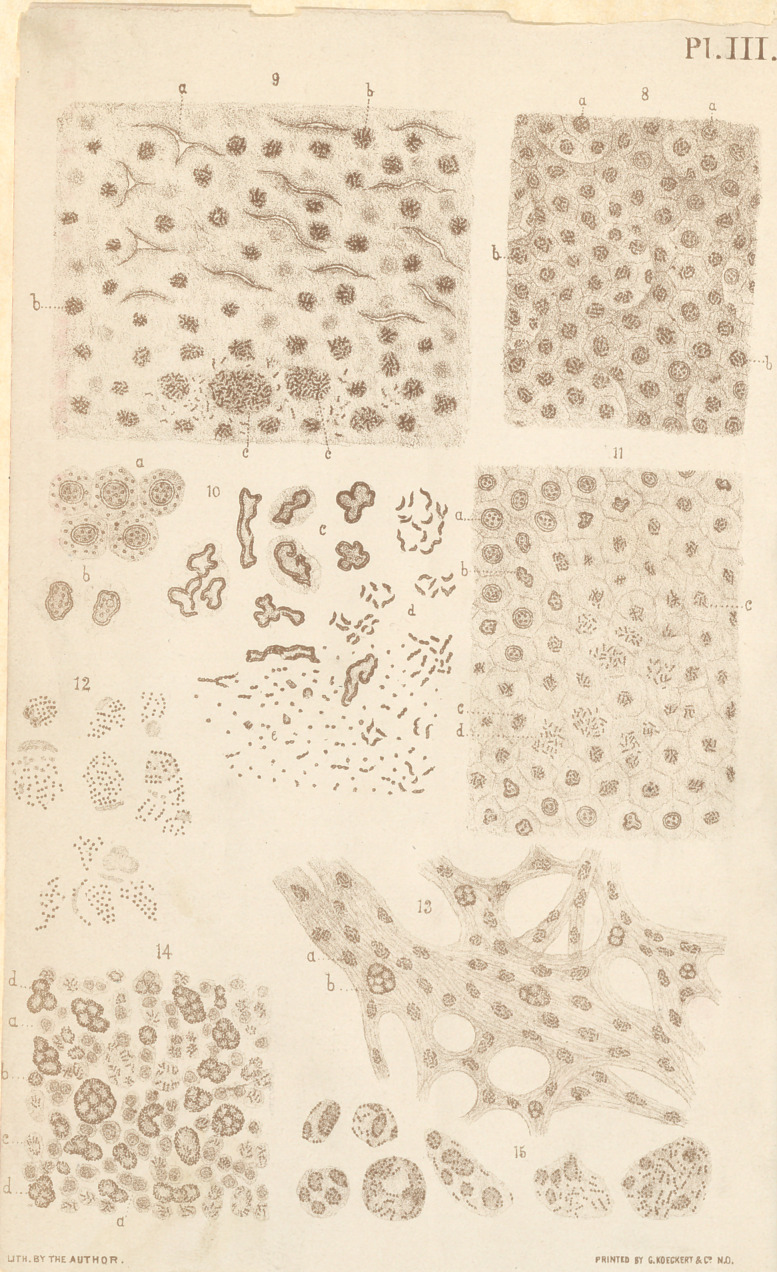# The Relationship of the Bacterium Tuberculosis to the Tubercles of the Human Lungs and Other Organs

**Published:** 1886-07

**Authors:** H. D. Schmidt


THE CHICAGO

### Medical Journal and Examiner.

Vol. LIII. JULY, 1886. No. 7.

### Original Communications.

V.

### The Relationship of the Bacterium Tuberculosis to the Tubercles of the Human Lungs and Other Organs.

The bacterium tuberculosis does not present itself in the same form, or in the same stage of development, in all portions of tuberculous lung, or even in all forms of tubercle. On the contrary, its development depends to a certain extent on the particular development, degeneration, and death of the tubercular growth. Greatest in number and highest in form of development, the organism is generally met with in tubercles which have very slowly and completely undergone the coagulation necrosis, and in which the outlines of the tubercle cells, as well as those of their nuclei, have disappeared. Such tubercles are generally met with in the lungs of cases of chronic tuberculosis. In some of these tubercles the bacteria tuberculosis are met with, embedded in the degenerated tissue, in very large numbers, whilst in others

W1
CH562

they are not so numerous. They are always found to lie in groups of four or five, or even more individuals, in the same way as they are frequently met with in the tuberculous expectorations. The morphological characters of the bacteria contained in these tubercles are the same exhibited by those already described and met with in tuberculous sputum. In examining microscopically thin sections of tuberculous tissue, containing very numerous bacteria tuberculosis, only a certain number of them can be at one and the same time brought into focus and distinctly seen in their proper outlines; the rest, being embedded more deeply in the tissue, and out of focus, will cause the tissue itself to appear as if stained by the same color, which in reality is not the case. Although the peculiar grouping of the bacteria tuberculosis has also been observed and mentioned by other authors, I have not, as yet, met with a satisfactory explanation for this phenomenon. It appears, therefore, that it is generally regarded as a matter of fact which needs no special explanation, if we simply regard the individuals, forming one group, as the descendants of that particular bacterium which, coming from the surrounding atmosphere, first entered the lungs during the act of respiration, to settle on the very spot in one or the other alveolus, where the group is found. And I must confess that in the beginning of these investigations, I entertained to a certain extent a similar view, until I made a certain observation, which explained to me the phenomenon of the bacteria tuberculosis appearing almost always in the form of groups, when met with in the tissues of the human organism. This observation I shall now describe.

In the latter part of the spring of 1883, after I had successfully stained the bacterium tuberculosis in tuberculous expectorations, and in numerous thin sections made from different tuberculous lungs, and, while still testing different methods of staining, I observed in sections, stained with methyl violet, and decolored by alcohol, that, though no stained bacteria could be discovered in the section, a number of nuclei had retained some of the blue color, while the rest of these bodies had given it up. At the same time, I noticed in the interior of these nuclei several granular bodies of a slightly deeper stain. Regarding these stained nuclei as having been formed more recently than those unstained, and the bodies which they contained as their ordinary, legitimate granules, I attached no particular importance to this observation. Some time afterward, however, when again examining sections, made from the lungs of a case of typical miliary tuberculosis, and stained by Gibbes' method, first with magenta and then with chrysoidine, I observed the same phenomenon, though more distinctly than before. In these sections, in which the tubercles were comparatively small, and in which no bacteria tuberculosis whatever could be discovered in their cheesy degenerated centres, I observed a number of deeply stained granules, as well as granular filaments, in the interior of the nuclei of the tubercle-cells occupying the periphery of the tubercle. Whilst these bodies, which in every respect resembled bacteria, were deeply stained by the purplish blue of the magenta, the protoplasm of the nucleus itself was stained yellow by the chrysoidine. This observation explained to me at once not only the cause of the peculiar grouping of the bacteria tuberculosis, as observed in the cheesy parts of the tubercles, but, moreover, their true origin in the nuclei of the tubercle-cells, and also showed me the direction in which my investigations had to be further pursued. In examining other sections of the same lung, stained in the same manner, I then observed the same purplish blue bodies not only in the interior of the nuclei of the tubercle-cells, but also in the nuclei of all the cells contained in the different tissues of the bronchioles adjacent to tubercles; that is, in the nuclei of the connective tissue-cells of the mucous and fibrous coats (fig. 6 *a*) in those of the muscular fibre-cells (fig. 6 *b*) and even in those of the cartilage-cells contained in the minute cartilaginous plates of the bronchioles. I furthermore observed these bodies in the nuclei of the cells of the adventitia of some of the smaller arteries. By repeatedly observing this same phenomenon afterward in sections made from the tuberculous lungs of other cases, especially of miliary tuberculosis, I was, at last, convinced of the bacterial nature of these stained bodies within the nuclei of the bronchial walls; the more so as they were not observed in the nuclei of the walls of bronchioles not adjacent to tubercles, nor in those of the healthy parenchyma of the lungs. While these bodies were stained of a deep purple by the magenta, the protoplasm of the nuclei appeared stained more feebly by the same color, indicating that the latter were affected by some irritation, probably derived from the neighboring tubercle. In staining these sections with a second color, such as chrysoidine or Bismarck brown, it was absorbed by the protoplasm of the nuclei, while the bacteria within the latter kept their purple tint; only when exposed a comparatively long time to these second colors, the bacteria assumed a brownish tint, though much darker that that of the nuclear protoplasm.

The correctness of the observations just described, I found corroborated by the examination and study of a very considerable number of stained sections, taken not only from the lungs of cases of miliary, but also of chronic tuberculosis, and of tuberculous livers, spleens and lymphatic glands, in which the same well-stained granular bodies were always observed in the interior of the nuclei of those tubercle cells occupying the periphery of the tubercle. All that now remained to prove the bacterial nature of these bodies was to meet with such sections of tuberculous tissue in which not only the origin and development of the bacteria tuberculosis in or from the protoplasm of the nuclei, but also their liberation from the latter could be clearly demonstrated. By the close examination of numerous well stained sections of large miliary tubercles of the lungs, liver and spleen, or of such tubercles situated near the borders of the tuberculous portions of the lungs of cases of chronic tuberculosis, I succeeded, at last, in tracing in one and the same section the bacterium tuberculosis from its first appearance in the nuclei of the tubercle cells to its full development and its liberation from the degenerated nuclei in the cheesy centers of the tubercles. Specimens of tubercles in which the bacterium tuberculosis can be traced from its origin to its liberation are, for certain reasons, to be stated directly, not found in every tuberculous portion of lung, liver, etc., though they are often enough met with, especially if properly looked for, to furnish to every investigator ample opportunity to corroborate the correctness of my statements.

As far as I know from the current medical literature it has now been asserted by a number of investigators on the subject under discussion, that free bacteria tuberculosis *cannot be discovered in all tubercles*, and, moreover, that whenever they are met with in tuberculous tissues, it is principally in that portion of tubercle, or tuberculous mass, in which the process of coagulation-necrosis is going on. My own observations not only corroborate the truth of the statements of these authors, but also enable me to furnish in the following pages the explanation for this phenomenon.

From my observations it appears that the development of the bacteria tuberculosis in the nuclei of the tubercle cells stands in a certain relationship with the degree or rapidity of the degenerative process taking place in the nuclei; in other words, the more rapid the course of the degenerative process, —not only of the nuclei but also of the protoplasm of the cells,—the smaller is the chance for the bacteria to reach their full development or maturity; whilst on the other hand, the more slowly this process proceeds, the more time will be afforded to these organisms for reaching their full development and their final liberation from the degenerated protoplasm of the nuclei.

The appearance of the bacteria in the nuclei is always preceded by a shriveling of the nuclear protoplasm, indicating that the degenerative process has commenced. The normally round outlines of the nucleus, then, become irregular, whilst its double contour appears darker and more prominent. With the advancing degeneration the irregularity of the outlines of the nucleus increases, whilst its margin, represented by the double contour, is breaking up, first into a few and then into more fragments, which are finally converted into a number of minute filaments of a granular or bead-like composition. In the course of the degenerative process the power of retaining aniline colors against decolorizing agents, such as nitric or formic acid, etc., increases in the margin of the nucleus, and in the fractions into which it has broken up, in proportion to the degree of degeneration. It is thus that in thin sections of tuberculous tissue, stained with magenta, the irregular shriveled margins appear of a dark red, which ultimately, in the filamentous fragments changes into purple.

Whilst the above described changes are observed to take place in the protoplasm of the nuclei, that of the cells, of course, undergoes the same degeneration. Accordingly the bodies of the cells appear paler and paler, until finally their outlines can be no longer distinguished, their protoplasm having lost all power of absorbing coloring materials, and undergone the coagulation-necrosis. As far as I am able to judge, the degenerative process not only commences in the protoplasm of the cells, but runs there a more rapid course than in that of the nuclei. This is seen in examining the cheesy centres of miliary tubercles, in which no trace of the outlines of the cells can be discovered, while a number of shriveled nuclei, containing forms of more or less perfectly developed bacteria, or minute fragments of nuclei, are still met with. Judging, however, from the fact observed in these cases, that the remains of the protoplasm of these nuclei still present the yellow color of the chrysoidine, or Bismarck brown with which the section was lastly stained, whilst the bacteria show the purple of the magenta, it appears to me that the protoplasm of the nuclei never undergoes a perfect degeneration, as, otherwise, it would not have retained its capacity of absorbing coloring matters. It seems, therefore, more probable that the shriveling of the nucleus stands in some relation to the development of the bacteria, for the more fully the latter are developed, the more the nucleus shrivels; in other words, the protoplasm of the nucleus appears to be necessary and appropriated for the development and growth of the bacteria.

In referring to some of the illustrative figures, accompanying this treatise, the reader will be enabled to understand more fully my statements regarding the development of the bacteria tuberculosis in the nuclei of the tubercle cells. Let us begin with fig. 3, which represents a small part of the periphery of a miliary tubercle with several of the neighboring non-tuberculous alveoli magnified about 625 diameters. In this figure we recognize at *a* the shriveled nuclei containing more or less developed bacteria tuberculosis, at *b* the epithelial cells of the non-tuberculous alveoli, and at *c* the interalveolar septa with the nuclei of their multiplied connective tissue-cells derived from the adventitia of their vessels, etc.; *d* represents a small portion of the periphery of the tubercle, while *e* represents a young tubercle which has arisen from a part of the wall of a large alveolus, or perhaps infundibular cavity, and which, contrary to the rule generally observed, has undergone the coagulation-necrosis at the periphery of the tubercle. The cheesy centre of the tubercle is situated toward the left and outside of the drawing. In proceeding with the examination of the anatomical elements represented in this figure from the right to the left side, we observe in the non-tuberculous alveoli, on the right side, already a number of nuclei which present the degenerative changes, above described, in their different degrees. Some of these nuclei are contained in the epithelial cells, while others appear to be located in the open spaces between these cells, occupied by the capillaries. From this it appears that the development of the bacteria tuberculosis not only takes place in the nuclei of the true tubercle cells, derived from the cells of connective tissue, but also in those belonging to the cells, lining the alveolar walls, and which descend from the cells of the entoderma. The same phenomenon is observed in the liver as we shall see hereafter. Although no trace of tubercular growth is found in these alveoli bordering the tubercle, quite a number of nuclei containing developing bacteria are nevertheless already observed in the inter-alveolar septa. The changes observed in these nuclei are most probably caused by a morbid irritation, derived and radiating from the neighboring tubercle. In passing to the left of the figure we observe at *d* the periphery of the tubercle. Here, most of the nuclei of the tubercle cells contain bacteria tuberculosis in different stages of development; the shades between these nuclei represent others out of focus. Finally at *e* we observe in the young tubercle, having already undergone the coagulation-necrosis, still a number of nuclei containing bacteria, together with others already disintegrated, the remains of which are observed throughout the cheesy mass in the form of fragmentary granular bodies. Although in this minute cheesy tubercle bacteria tuberculosis are distinctly seen in the remaining nuclei of tubercle cells, none of these organisms are met with entirely liberated from the nuclear protoplasm, either single, or forming larger groups, or aggregated in masses. On the contrary, after having reached a certain degree of development, they degenerate, die and disentegrate with the anatomical elements of the tubercle. As this condition of things is mostly found to exist in the smallest tubercles of typical cases of miliary tuberculosis, it explains the fact, observed and stated by some authors, that *not all tubercles contain bacteria tuberculosis*, and that, whenever they are met with, it is generally in the cheesy matter of old tubercles. I have microscopically examined the lungs of a fair number of cases of miliary tuberculosis, without even being able to discover free and fully developed bacteria tuberculosis in the smaller or younger tubercles of these organs; whenever they were met with, it was always in the older and larger tubercles. In the miliary tubercles of the liver and spleen, on the contrary, I have not unfrequently encountered them liberated from the nuclei, and fully developed; in these cases, however, the lungs always contained small cavities, showing that the course of the disease was rather protracted.

In directing now our attention to figure 4 which represents a portion of a small miliary tubercle near its cheesy centre, magnified about 1,090 diameters, we observe the same condition as in fig. 3, with the exception that the anatomical elements, being more highly magnified, are more distinctly recognized. As in the preceding figure, we observe at *a* the bacteria tuberculosis developing in the nuclei of the tubercle cells; the outlines of these cells are not seen, on account of the object being mounted in Canada balsam and illuminated very highly with Abbé's illuminating apparatus. At *b* the same nuclei are seen, but out of focus, while *c* represents the inter-alveolar septa. Finally at *d*, a portion of the cheesy centre of the tubercle is represented, in which the disintegration of the nuclei and bacteria tuberculosis is distinctly seen.

The degeneration of the nuclei and imperfect development of bacteria tuberculosis is equally well exhibited in figure 10, representing some of the anatomical elements of a miliary tubercle of the liver, magnified 1,090 diameters. In this figure, we observe at *a* a group of normal hepatic cells from near the border of the tubercle; *b* represents a pair of hepatic cells from the periphery of the tubercle, the nuclei of which have commenced to shrivel, while at *c* a number of nuclei from the cheesy centre of the tubercle are exhibited, the margins of which are shriveled in a higher degree and broken up into fragments. Some of these nuclei are still surrounded by the degenerating protoplasm of their cells. At *d*, finally, the ultimate minute fragments of the nuclei are represented, some of which have assumed the form of granular filaments.

In the cheesy centres of the older and larger tubercles, or tuberculous masses, met with in the lungs of cases of chronic tuberculosis, as well as in those of the larger tubercles found in the organs of protracted cases of miliary tuberculosis—especially when accompanied by the formation of small cavities in the lungs — the bacteria tuberculosis become fully developed and are liberated from the remaining protoplasm of the nuclei by its final complete degeneration. Although the gradual development of these bacteria, from their first appearance in the nuclei of the tubercle cells to their final liberation, and to their aggregation in larger groups, or masses, is not seen in the sections of every one of these tubercles, there are, nevertheless, many specimens met with—especially when looked for in the examination of very numerous sections—in which the whole process may be observed in one and the same section. It is from such a specimen that figure 5 was copied. This figure, also magnified 1,090 diameters, represents a small portion of a large tubercle from the lungs of a boy, sixteen years old, and affected with miliary tuberculosis of the lungs, liver, spleen, kidneys, and lymphatic glands; the lungs, from which the section was taken, contained several small cavities. The tubercle, though it had undergone the coagulation-necrosis, showed no cavity in the centre. In this figure, as in the preceding, *a* represents the bacteria tuberculosis developing in the nuclei of the tubercle cells, and *b* the same out of focus. At the upper portion of the figure, the borders of two neighboring alveoli are recognized, while *c* very likely represents some degenerated interstitial connective tissue, leading to the central part of the tubercle. In the latter we observe at *d* the bacterial groups commencing to enlarge in their dimensions, while at *e* we find them aggregated into small masses. In the sections, the latter present a purplish blue appearance, caused by those bacteria being buried deeper in the tissue, and in consequence out of focus.

Though I am as yet unable to assert anything positive as to the exact manner in which these aggregations of bacteria tuberculosis are formed, I think that it may take place as follows: When the bacteria contained in one nucleus have reached their full development, they may be liberated by the complete degeneration and disintegration of whatever has remained of the protoplasm of this nucleus, and then multiply in the protoplasm of the body of the cell, provided it has as yet not completely undergone the necrotic process. I doubt not but that in this manner the groups of bacteria, originally contained in the nuclei, frequently grow in dimension, and that this mode of growth has given rise to the idea, generally entertained, of these organisms being contained in the protoplasm of the tubercle cells. The groups, represented at *d* of figure 5, may have grown in this way. The larger aggregations of bacteria, represented at *e*, may have been formed in the same manner, that is, by a complete necrosis and fusion of the protoplasm of a number of neighboring cells, together with a numerical increase of the bacteria contained in these cells or their nuclei. The formation of these aggregations or masses of bacteria tuberculosis in the manner just indicated is very probable, for the reason that by a close microscopical examination the original groups, forming these masses, may in many instances be still recognized.

In some of the miliary tubercles of the liver the development of the bacteria tuberculosis in the nuclei of the cells as well as their liberation from the latter and their aggregation into small masses is frequently well exhibited in one and the same section. In the preceding division of this treatise I have already mentioned that the secreting elements of the liver, the hepatic cells, appear to take a part in the formation of the tubercle; at any rate, bacteria tuberculosis are observed to develop in the nuclei of these cells. To illustrate this fact let us examine figure 8, which has been copied from a section of the liver of the boy of sixteen years, above mentioned, and represents a small portion of the parenchyma of this organ, bordering directly the periphery of the miliary tubercle, magnified about 625 diameters. In this figure we observe a number of hepatic cells (*a*) the nuclei of which still present a normal appearance, whilst in others the degenerative changes of these bodies, with the gradual development of the bacteria tuberculosis (*b*), are distinctly seen. In passing over the figure 9, representing a portion of the centre of the tubercle which has undergone the coagulation-necrosis—also magnified 625 diameters,—we observe a number of fissures (*a*) in the section which, very likely, represent the contracted lumen of the capillaries. At (*b*) the individual groups of bacteria tuberculosis, with the remains of the protoplasm of the nuclei in which they have been developed are distinctly seen. In passing from the upper to the lower part of the figure the bacteria of these groups are observed to increase in number until at (*c*) they appear as smaller or larger aggregations.

During the course of the development of the bacteria tuberculosis in the nuclei of the tubercle cells they appear both in the form of cocci or diplococci and of filaments composed of three, or even four cocci. The latter appear generally curved, and frequently lie parallel with the periphery of the nucleus, so that in the beginning of their formation they might be mistaken for the double contour of this body. That this is not the case, however, is easily discovered by observing the granular fragments into which the contour is broken up. The single granules, or cocci, do not represent the normal granules contained in the nuclei, as might be thought; this is proved by their retaining the purple color of the magenta when subjected to the action of decolorizing agents, a property which the latter do not possess. Besides the normal granules of the nuclei occupy the interior and not the periphery of these bodies. But, as not all the nuclei of the tubercle cells contain bacteria, the difference between these organisms and the normal organic granules is easily recognized by the latter being stained, like their nuclei, with the second color of the section.

In sections made of tuberculous lung tissue bordering a smaller or larger cavity, the gradual development of the bacteria tuberculosis may also be frequently observed. In the tuberculous tissue, forming the walls of the cavities, which is generally more or less indurated, the bacteria may still be contained in the remains of the protoplasm of the nuclei, whilst at the very borders of the cavity, as well as in the layer of inspissated purulent matter, by which the latter is generally lined, groups of free bacteria may be met with. By the breaking down of the tuberculous tissue the bacteria get into the purulent matter contained in the cavity, and with this into the expectorations of the patient. Not in every instance, however, are bacteria tuberculosis met with in the sections taken from the borders of pulmonary cavities, a circumstance which explains the fact of the occasional absence of these organisms from the expectorations of tuberculous patients.

The development of bacteria in the protoplasm of the nuclei of irritated and degenerating cells, however, is not only observed in tissues affected by tuberculosis, but also, as I shall show directly, in the neoplastic degenerating cells of organs affected by other diseases, such as leprosy, carcinoma, etc.

In the same manner as tuberculosis is characterized by the presence of the bacterium tuberculosis in the tubercles of the organs affected by this disease, leprosy is characterized by the so-called Bacillus leprae. This organism, though called a bacillus, presents itself still more distinctly in the form of very minute cocci than the bacterium tuberculosis. The chief difference found between the two organisms consists in the cocci of the bacterium of leprosy being smaller than those of the bacterium tuberculosis, as well as in the filaments formed by these cocci being almost always straight. With the view of closely investigating the morphological characters of the bacillus leprae, I amputated in the summer of 1883 the lobuli of the ears of two living leprous patients. The examinations made on the material thus obtained were not only confined to thin sections, but extended to the fresh blood oozing from the cut vessels, and to the matter scraped with a sharp knife from the freshly cut surface of the lobules. All the preparations were carefully stained by Gibbes' method with magenta and chrysoidine. In the matter scraped from the cut surface of the lobuli I met, as I had expected, with single leprous cells, detached from the connective tissue of the skin by the scraping process, which contained single cocci, diplococci, and filaments composed of three or four cocci, representing the bacillus leprae.

In the stained preparations which I had carefully made of the blood oozing from the cut vessels of these lobuli, I discovered the same organisms, showing the morphological characters, above mentioned, still more distinctly than in the leprous cells. They were here observed attached in the form of groups to the colorless blood corpuscles, the number of which appeared to be abnormally increased. The protoplasm of most of these corpuscles had undergone degeneration and disintegration, the bacteria appearing liberated, or adhering to the remains of the likewise degenerated nuclei (fig. 12, magnified about 1090 diameters). Subsequently to this observation a number of other preparations were made of leprous blood, taken from different parts of the skin of three leprous patients by my young friend Dr. E. Laplace, at present sojourning in Paris, who was, at that time, the student assistant of the Pathological Department of the Charity Hospital. In all these preparations the same organisms were found, exhibiting the characters above described. Although a number of opportunities were offered to me, since that time, to examine the blood of other lepers, I neglected to do so for the want of time; until during the summer of 1885, when I again examined the blood taken from the arms of two other leprous patients, without meeting, however, with any bacteria contained in this fluid.

The same form of bacteria as those met with in the blood were observed in the protoplasm of the cells and their nuclei in the pus covering the healing surface of the ears, from which the lobuli had been cut. In the matter taken from a pustule of the skin of another leprous patient, a woman, the bacteria were met with mostly in the form of granular filaments in the nuclei of the leucocytes.

In the sections, made from the amputated lobules of the ears, I found, likewise, the bacteria developing in the nuclei of the neoplastic cells. But, as many of the cells contained in such a section had undergone fatty degeneration, and, in consequence, were filled with a number of smaller or larger fat globules, whilst at the same time they contained quite a number of nuclei, formed from the original nucleus by the process of division—and which eventually undergo degeneration— a careful examination is required to clearly understand the exact location of the bacteria in the larger kind of these neoplastic cells. For this reason our attention should be directed to those cells, of which the protoplasm has as yet not undergone fatty degeneration, and in which the multiplication of the nuclei has not advanced too far. In these cells the bacteria will be seen developing in the nuclei (fig. 14, *a*, *b*, and *c*); and, when closely examined with a good homogeneous immersion objective of a sufficient magnifying power, and illuminated with very oblique light, they will show that they are composed of minute cocci, appearing in bas-relief-like minute beads, and exhibiting the character of a true *sphero*-bacterium. In consideration of this fact I do not hesitate to call hereafter the so-called Bacillus leprae by its proper name, the *bacterium leprae.*

In the latter part of the spring of 1884, another fair opportunity of investigating the morphological characters of the bacterium leprae was offered to me by the death of a severe case of leprosy at the Charity Hospital. The microscopical examination of the tissues of the lungs, liver, spleen, kidneys, epiglottis, etc., fully corroborated the correctness of my previous observations concerning the bacterium leprae. In the liver and spleen the development of these bacteria in the muclei of the cells was particularly well exhibited, as will be seen by referring to figure 11. In this figure, which represents a small part of a thin section of the liver, stained by Gibbes' method, and magnified about 625 diameters, we observe at *a* the normal nucleus of a hepatic cell, while that of a neighboring cell, at *b*, shows the beginning of the degenerative process by the shriveling of its margins. In proceeding to *c*, we find the outlines of the nucleus obliterated and the bacteria leprae nearly developed; while finally at *d* they are seen completely liberated from the degenerated protoplasm of the nucleus and disseminated through the likewise degenerating protoplasm of the body of the cell.

Finally in the summer of 1885 I took occasion to convince myself once more of the correctness of my previous observations, by examining very thin sections made of a leprous tubercle cut from the arm of a living patient, and also stained by Gibbes' method. Small portions of one of these sections I have represented in figures 13, 14 and 15. The first of these (fig. 13) represents a minute portion of the pars reticularis of the corium of the skin from the very margin of the tubercle, where the pathological changes of the connective tissue cells are still in the incipient stage of the disease; it is magnified 625 diameters. In it, we observe a considerable number of the nuclei of these cells (*a*) distributed throughout the fragments of tissue containing bacteria leprae in their early stage of development. Of these cells, only a small number (*b*) are seen in an advanced stage of fatty degeneration with the fully developed bacteria leprae between the fat globules. In figure 14, which represents a minute part from the middle of the tubercle, and also magnified about 625 diameters, the bacteria are seen in the different stages of their development. Thus at *a* their first appearance is noticed in the nuclei of the unchanged connective tissue cells, whilst at *b* they are slightly more developed. At *c* the nucleus has disappeared by degeneration, and the bacteria are seen fully developed in the protoplasm of the cell, exhibiting very distinctly their grander character. Finally at *d* they are, as before, met with fully developed between the flat globules of the degenerated cells. In figure 15, which represents a number of degenerated leprous cells, magnified about 1090 diameters, the morphological character of the bacteria leprae is still better recognized than in the preceding figures.

In order to ascertain whether, like in the nuclei of the cells of tuberculous and leprous tissues, bacteria would also develop in those of other morbid growths, I examined during the course of the above described investigation, the sections of quite a number of fresh tumors, or other neoplasms, stained by Gibbes' method. Although in the greater number of these specimens bacteria were not met with, I nevertheless found my suspicions verified in distinctly observing in four instances the development of these organisms in the nuclei of the neoplastic cells in the same manner as I have described it above. The first of these instances relates to a small lobulated tumor, about 5/8 inch in diameter, a so-called polypus of the mucous membrane of the rectum, removed from very near the verge of the anus. In examining the sections of this tumor, I met in some places of the hyperplastic connective tissue a large number of cells the nuclei of which contained granular bodies resembling bacteria. That these bodies in reality represented bacteria, was proved by their being intensely stained purple, or purplish blue, by the magenta, while the protoplasm of the nuclei was stained yellow by the Bismarck brown. They presented themselves mostly in the form of cocci, or diplococci, but there were also some filaments observed, composed of three cocci. The diameter of these organisms was about the same as that of the bacteria tuberculosis, though among them a number of single cocci of a still larger diameter were noticed. In the nuclei of a small number of the deeper cells of the epithelium covering this tumor, as well in some of the Lieberkuehn's glands, the same bacteria were met with. Although the sections of this tumor were made, stained and mounted in Canada-balsam in January, 1884, the bacteria were seen as distinctly as ever when re-examined in September, 1885, though the purplish blue of the magenta had faded into a dark red. The next specimen, in which I met with bacteria in the nuclei of the cells, was a carcinoma of the mammary gland. There they also appeared in the form of cocci and diplococci, but, as in the preceding case, only in certain places of the section. In both instances the bacteria were strictly confined to the protoplasm of the nuclei.

In the third specimen, however, representing a carcinoma of the stomach, the bacteria were observed to develop in the same manner as those in the nuclei of the cells of tuberculous or leprous tissues, that is, commencing with the shriveling of the nucleus, and the breaking up of the margin of this body into minute fragments, and terminating in the formation and liberation of granular filaments. In this case also, the bacteria were only met with in certain localities of the section, especially in the cells of the fibrous coat of the organ. In the fourth morbid growth, representing a so-called endothelial cancer, the whole process of development could also be traced from the shriveling of the nucleus to the ultimate formation and liberation of the bacteria, which, in some places, were met with aggregated in the form of small groups. Some of these groups were formed by three or four filaments lying at certain angles toward one another; sometimes in the form of a circuit, resembling a ring broken into fragments, and corresponding in size to the nuclei in which they had developed. Most of the filaments were more or less bent or curved.

The bacteria met with in these two last neoplasms did not exhibit the deep purplish-blue, observed on the fully developed bacteria tuberculosis and leprae when stained with magenta, but rather a purplish red. This want of deepness in the stain was undoubtedly owing to their not having as yet reached their highest development. This same phenomenon is frequently observed during the development of the bacterium tuberculosis in the nuclei of the tubercle cells, as I have once mentioned before.

The results of my examinations of these four neoplasms then show, that the development of bacteria in the nuclei of certain cells does not exclusively take place in tuberculous and leprous tissues, but also in other morbid growths, whenever the special conditions required for their evolution are present. What these conditions are, would be difficult to determine at our present state of knowledge, though it is not improbable that they may be found in a certain form of degeneration of the elements of these tissues, such as the coagulation-necrosis, which cannot be said to be confined to the tubercle of tuberculosis. The tumors, of which I stained sections for the purpose of discovering bacteria, though representing a fair number, are far from comprising all those which I examined during the course of these investigations, as, otherwise, the number of specimens in which bacteria were met with might, perhaps, have been larger. But as the time which I could spare for the search of bacteria in other than tuberculous and leprous tissues was rather limited, I confined my examinations only to specimens which presented certain phenomena of involution or decay, and contented myself with the four instances above mentioned.

## Figures and Tables

**Pl. III f1:**